# Bats that walk: a new evolutionary hypothesis for the terrestrial behaviour of New Zealand's endemic mystacinids

**DOI:** 10.1186/1471-2148-9-169

**Published:** 2009-07-20

**Authors:** Suzanne J Hand, Vera Weisbecker, Robin MD Beck, Michael Archer, Henk Godthelp, Alan JD Tennyson, Trevor H Worthy

**Affiliations:** 1 School of Biological, Earth and Environmental Sciences, University of New South Wales, Sydney 2052, Australia; 2 Cambridge University, Department of Earth Sciences, Downing Street, CB2 3EQ, UK; 3 Institut für Spezielle Zoologie und Evolutionsbiologie, Friedrich Schiller-Universität Jena, Am Fürstengraben 1, 07743 Jena, Germany; 4 Museum of New Zealand Te Papa Tongarewa, Natural Environment Department, PO Box 467, Wellington, New Zealand; 5 School of Earth and Environmental Sciences, University of Adelaide, Adelaide 5005, South Australia, Australia

## Abstract

**Background:**

New Zealand's lesser short-tailed bat *Mystacina tuberculata* is one of only two of c.1100 extant bat species to use a true walking gait when manoeuvring on the ground (the other being the American common vampire bat *Desmodus rotundus*). *Mystacina tuberculata* is also the last surviving member of Mystacinidae, the only mammalian family endemic to New Zealand (NZ) and a member of the Gondwanan bat superfamily Noctilionoidea. The capacity for true quadrupedal terrestrial locomotion in *Mystacina* is a secondarily derived condition, reflected in numerous skeletal and muscular specializations absent in other extant bats. The lack of ground-based predatory native NZ mammals has been assumed to have facilitated the evolution of terrestrial locomotion and the unique burrowing behaviour of *Mystacina*, just as flightlessness has arisen independently many times in island birds. New postcranial remains of an early Miocene mystacinid from continental Australia, *Icarops aenae*, offer an opportunity to test this hypothesis.

**Results:**

Several distinctive derived features of the distal humerus are shared by the extant *Mystacina tuberculata *and the early Miocene Australian mystacinid *Icarops aenae*. Study of the myology of *M. tuberculata *indicates that these features are functionally correlated with terrestrial locomotion in this bat. Their presence in *I. aenae *suggests that this extinct mystacinid was also adapted for terrestrial locomotion, despite the existence of numerous ground-based mammalian predators in Australia during the early Miocene. Thus, it appears that mystacinids were already terrestrially-adapted prior to their isolation in NZ. In combination with recent molecular divergence dates, the new postcranial material of *I. aenae *constrains the timing of the evolution of terrestrial locomotion in mystacinids to between 51 and 26 million years ago (Ma).

**Conclusion:**

Contrary to existing hypotheses, our data suggest that bats are not overwhelmingly absent from the ground because of competition from, or predation by, other mammals. Rather, selective advantage appears to be the primary evolutionary driving force behind habitual terrestriality in the rare bats that walk. Unlike for birds, there is currently no evidence that any bat has evolved a reduced capacity for flight as a result of isolation on islands.

## Background

Only two of c.1100 extant bat species use a true walking gait when manoeuvring on the ground – the lesser short-tailed bat *Mystacina tuberculata *of NZ, and the common vampire bat *Desmodus rotundus *of Central and South America [1]. *Mystacina tuberculata * is the sole surviving member of Mystacinidae, which is the only living mammalian family endemic to NZ, although its distribution once included Australia [2]. A second NZ species of *Mystacina *(*M*. *robusta*) has become extinct c.1967 [3]. Exactly when and from where mystacinids first colonized NZ is not yet clear, but early Miocene mystacinid fossils have recently been found in NZ [4,5] and middle Cenozoic Australia has been proposed as their probable source [4,6]. *Desmodus rotundus *is a member of the Central and South American family Phyllostomidae. Mystacinids and phyllostomids fall within the Gondwanan bat superfamily Noctilionoidea, but molecular divergence dates indicate that the two families diverged 41–51 Ma [7], and terrestrial locomotion appears to have evolved independently in *Mystacina *and *Desmodus*.

Today, *Mystacina tuberculata *populations are restricted to extensive areas of old-growth indigenous NZ forests dominated by *Podocarpus*, *Dacrydium*, *Agathis *and *Nothofagus *spp [8]. This bat spends more time on the ground than any other: up to 40% of its foraging time [9,10] is spent scurrying with rodent-like agility over tree branches and the forest floor using its broad, backwards-facing feet and thick-skinned wrists as points of contact with the substrate. When foraging under leaf litter, humus or snow, it folds its long ears and often disappears completely, re-emerging only sporadically [8,11].

As long-recognized [e.g. [8,9,12-18]], *M. tuberculata*'s consummate terrestrial habits are reflected in numerous adaptations in its postcranial skeleton including specializations of the wing, foot, leg, spine, and pectoral and pelvic girdles. When moving terrestrially, its wings are furled tightly in a protective leathery sheath-like portion of the plagiopatagium. Its reduced pro- and uropatagia enable free movement of its fore and hindlimbs respectively [14,15]. Unique secondary talons at the base of the thumb and toe claws of *M. tuberculata *increase grip on the substrate, as does a system of adhesive, gecko-like grooves in its soft, deeply-wrinkled pedal soles [12-15]. *Mystacina tuberculata *is an important pollinator of NZ's endemic parasitic wood rose, *Dactylanthus taylorii*, the world's only known ground-flowering plant to be pollinated by a bat [19].

### Previous hypotheses for the evolution of terrestriality in bats

It has previously been assumed that the specialized terrestrial habits of mystacinids evolved in NZ following their isolation there, just as flightlessness evolved rapidly and independently many times in island birds of NZ and elsewhere [e.g. [20-22]]. In the case of mystacinids, a lack of native terrestrial mammalian predators in NZ has been hypothesised to have facilitated evolution of terrestriality [e.g. [1,9,11,17]]. In the case of *Desmodus rotundus*, by contrast, the absence of mammalian nocturnal predators and/or competitors is not regarded as the driving force behind evolution of terrestrial locomotion; instead, it has been suggested that a running gait confers an energetic benefit and hence selective advantage by enabling *Desmodus *to chase prey that flee in the middle of a feeding event [1].

Until now there has been little opportunity to test hypotheses for the evolution of terrestrial locomotion in mystacinids. Recently, however, postcranial remains of an Australian early Miocene mystacinid have been recovered from the Riversleigh World Heritage Area (WHA), northwestern Queensland. In this paper we describe the distal humerus of this bat and examine the likely functional attributes of its elbow based on comparison with morphology of this joint and associated musculature in *Mystacina tuberculata*. We discuss the implications of our findings for the temporal and geographical origins of habitual terrestriality in mystacinids.

## Results

### Elbow morphology and implications for locomotion in *Mystacina tuberculata*

**Figure 1:**
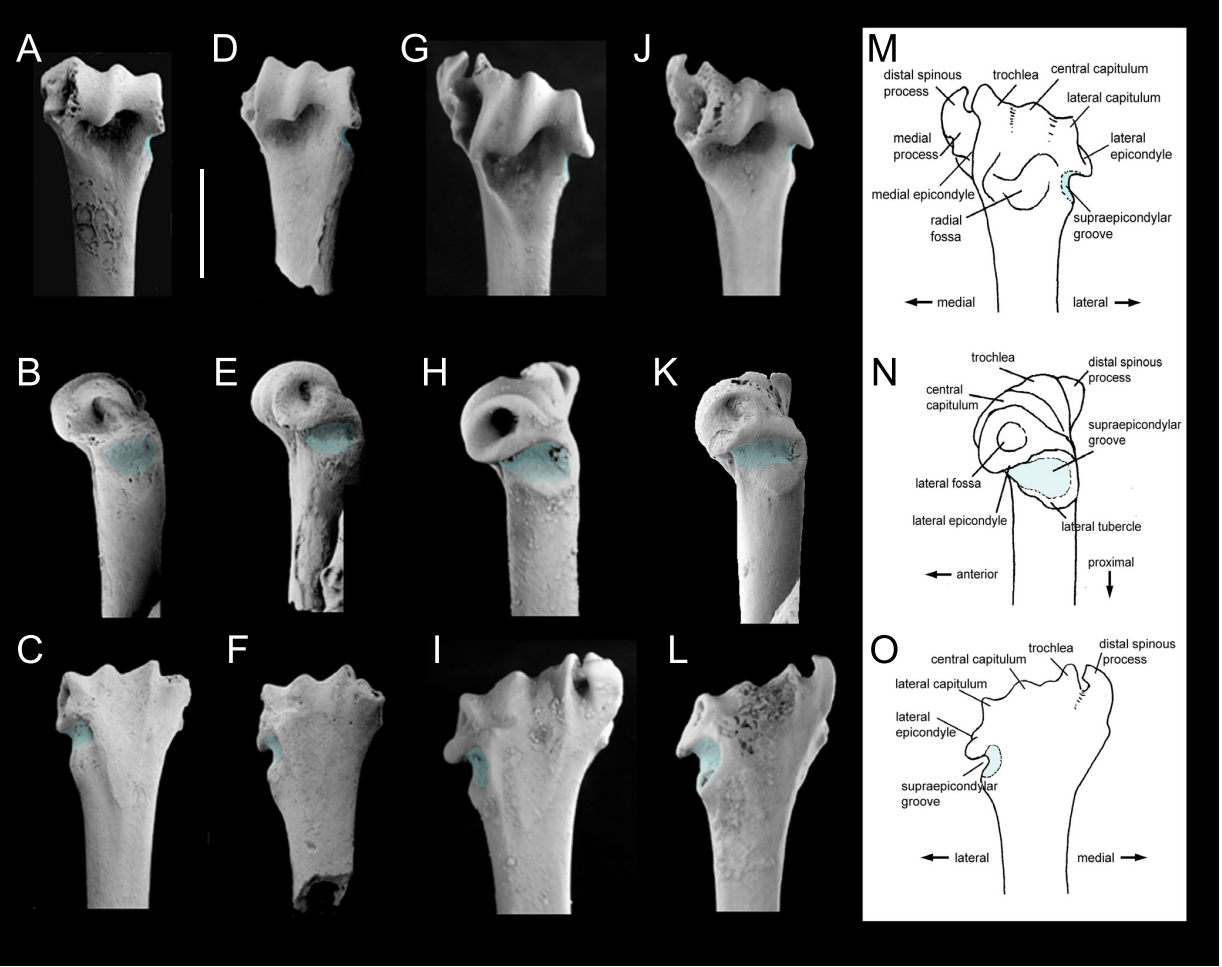
**Comparison of distal humerus morphology of Australian Miocene *Icarops aenae *with Recent NZ *Mystacina *spp**. **A-C**, *Icarops aenae *QM F30573, Wayne's Wok Site, Riversleigh WHA, Australia; anterior, lateral and posterior views (flipped). **D-E**, *I. aenae *QM F30574, View Delightful Site, Riversleigh WHA; anterior, lateral and posterior views. **F-H, *Mystacina robusta ***NZ S-35205, Exhale Air Cave, Ellis Basin, Mt Arthur, Nelson, NZ; anterior, lateral and posterior views. **I-L**, *M. tuberculata *NZ S-32400, Predator Cave, Takaka Hill, Nelson, NZ; anterior, lateral and posterior views. **M-O, terminology**: anterior, lateral and posterior views; medial process [e.g. [28]] = epitrochlea [e.g. [28]] = medial epicondyle [e.g. [32]]; distal spinous process [e.g. [28]] = spinous process [e.g. [32]]; central surface of capitulum [e.g. [28]]. Supraepicondylar groove shown in blue. Scale bar = 4 mm.

The morphology of the elbow in bats has long been recognized as a rich source of information, both systematic [16,23-29] and functional [27,28,30-33]. In particular, the morphology of the distal humerus has been widely used to infer flight mode and terrestrial capability in extinct and extant bats. In *Mystacina *species, the humeral capitulum is non-spherical (with its articular surface delimited laterally and medially by ridges and grooves) and its articular surface is mostly aligned with the shaft (Figure 1). This results in a relatively rigid humeroradial articulation that allows motion only in the anteroposterior plane, and is associated with relatively fast, direct flight [28,32,33].

However, the medial process (epitrochlea) of *M. tuberculata *is significantly broader than that of most fast-flying bats, and its separation from the trochlea and deep scars for muscle attachment suggest a relatively large muscle mass and hence the capacity for relatively more manoeuvrable flight [28]. Thus, humeral morphology in *M. tuberculata *appears to represent a trade-off between the demands for fast, direct flight and manoeuvrability. This is congruent with the suggestion of Webb *et al*. [34] that the wing morphology of *M. tuberculata *represents a compromise between different adaptive pressures: i.e. slow, manoeuvrable foraging flight within dense forest, and fast, direct commuting flight between forest patches.

**Figure 2:**
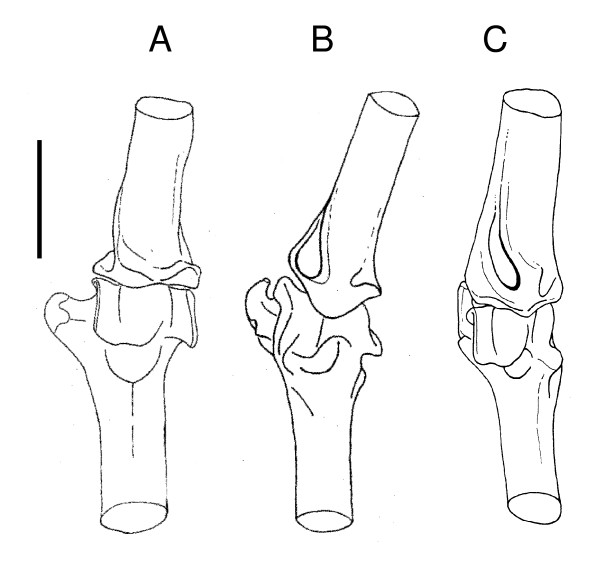
**Elbow morphology of three representative bats comparing degree of articulation**. Anterior view of the right humeroradial (elbow) articulation of: A, *Desmodus rotundus*; B, *Mystacina tuberculata*; C, *Molossus molossus *Most rigid articulation occurs in *Molossus *(C), least in *Desmodus *(A), with *Mystacina *(B) exhibiting a pronounced inclination in articulation which directs the radius laterally. (A and C, after Smith 1972 [28], Figures 3 and 5) Scale bar = 4 mm.

Unlike birds, bats use all four limbs for terrestrial locomotion [1]. Not surprisingly, chiropteran elbow morphology has previously been correlated with terrestrial agility as well as flight mode; extensive articulation between the trochlea and the radius characterizes not only bats that are fast, direct fliers but also those that are relatively agile on the ground [e.g. molossids and some vespertilionids; [18,32,33,35]]. However, this is not the case in bats that use a true walking gait, namely species of *Desmodus *and *Mystacina*. *Desmodus rotundus *retains (plesiomorphically) a spherical central capitulum and large medial muscle mass enabling considerable rotational (anteroposterior and lateral) movement in the elbow joint (Figure 2A). *Mystacina tuberculata*, on the other hand, shares with molossids, for example, a more derived precise and restrictive bony articulation between the humerus and radius (Figure 2B, C), but combines this with the specialized muscle morphology described below. The very different morphological and myological constraints on the elbow joint in species of *Desmodus *and *Mystacina *is consistent with independent evolution of a quadrupedal walking gait in these two lineages as proposed by Riskin *et al*. [1].

The humerus of *M. tuberculata *is additionally characterized by a conspicuously laterally inclined humeroradial articulation (Figures 1, 2B). This humeral specialization results in a distinctive movement of the radius during walking because it causes the radius to move in a laterally-directed arc. Video footage [36] and treadmill frames [[1], Figure 1C, F] of terrestrial locomotion by *M. tuberculata *confirm this observation. The humerus is held closely adducted and parallel with the long axis of the body, and the radius moves in a plane away from the humerus and body, never actually coming to lie beneath the body as it does in most quadrupedal mammals. The characteristic lateral inclination of the trochlea directs the radius laterally during the stride and pushes the body sideways and forward, resulting in a scuttling rather than striding movement. With the body held close to the substrate, this style of locomotion is well suited to moving in confined places, and presumably also for digging through and under leaf litter.

The distal spinous process of *M. tuberculata *is characteristically elongate and represents the distalmost extremity of the humerus. The direction of flexor muscle action differs depending on the position of the radius because the distal position of the spinous process shifts the flexors from the centre of rotation. When the elbow is flexed, the distal spinous process forms a wide angle with the radius so that the distance between the process and the distal wing is greater. Consequently, the mechanical advantage accrued by the muscles for flexing the hand is also greater It is possible that during terrestrial locomotion (when the elbow is half-flexed) the greater distance and moment arm improves flexion power of the muscles operating the distal carpus which remains flexed. This is congruent with the general tendency for the extensor and flexor muscles of the chiropteran hand to act as inelastic cords that automatically move the manus with movements of the forearm in flight [32].

### Myology and functional morphology of the humerus of *Mystacina*

**Figure 3:**
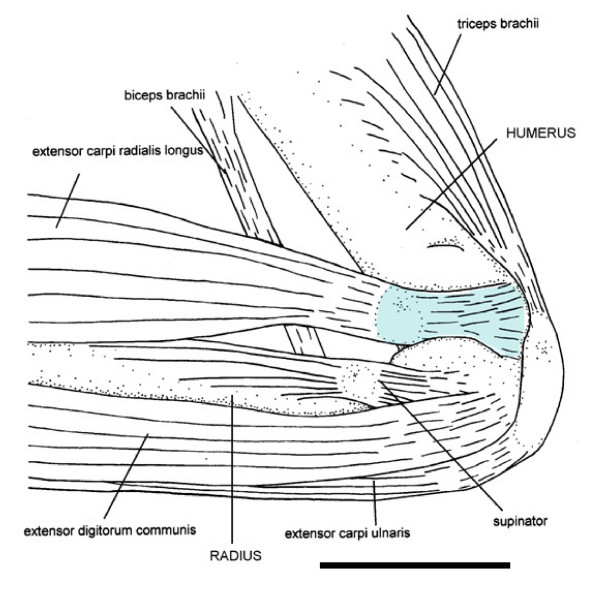
**Muscular origins and insertions of the distal humerus of *Mystacina tuberculata***. Schematic diagram of the muscular origins and insertions of the distal humerus of *Mystacina tuberculata *(NMNZ LM 1231, Kaikohe, Northland, NZ). The *M. extensor carpi radialis longus *(ECRL) is shown lifted from the radius to better view its sesamoid. Blue shading indicates ECRL course in supraepicondylar groove of distal humerus. Scale bar = 4 mm.

Myological examination of the forelimb of *Mystacina tuberculata *reveals that the morphology of the humerus reflects specializations in muscular morphology and presumably therefore muscle action. Dissection of the elbow of *M. tuberculata *(NMNZ LM 1231) shows that the characteristic deep supraepicondylar groove proximal to the lateral epicondyle (Figure 1) is occupied by the tendon of the large *M. extensor carpi radialis longus *(ECRL; Figure 3). This muscle originates in a strap-like tendon from the posterolateral rim of the groove. The tendon extends across the lateral surface of the humerus and attaches to the tubercle on the lateral side of the distal humerus, directly proximal and slightly posteromedial to the deep tendon groove. As such, the ECRL tendon inserts markedly medially. A wide portion of the ECRL tendon is lodged within the humeral groove; anterior to the groove, the body of this tendon houses a sesamoid. The tendon leaves the groove through the characteristic cleft before entering a conspicuously large muscle. The flexor muscles of the carpus and digits and the *pronator teres *muscle originate on the medial rim of the distal spinous process. Their medial origin is displaced distally, which results in a considerable distance between the flexor muscle origins and the radius, resulting in a larger moment arm for the flexor muscles.

The ECRL position, with its tendon housed in the conspicuous lateral supraepicondylar humeral groove, is unique among bats. The origin of the ECRL winds around the lateral side of the humerus and attaches to the posteromedial tubercle; thus, the origin of the ECRL is more medial than in other bats which may give the muscle slightly more leverage on the humerus. The ECRL inserts on the dorsal base of the first metacarpal and anterodorsal base of the second metacarpal which it directly extends [32,37,38] and acts indirectly to extend the entire distal part of the wing [38]. Because the ECRL is conspicuously large in *M. tuberculata *compared to that of other bats [e.g. [32,33,35,37,38]], its action should result in a powerful concomitant extension of the first and second metacarpal. The direction of this motion appears to be tightly constrained because of confinement of the tendon's origin in the deep humeral groove.

One possible function of this powerful arrangement could be to facilitate take-off. *Mystacina tuberculata *launches into the air from the ground by leaping from a quadrupedal stance rather than flapping its wings [34] thereby necessitating some levering action of the limbs. The powerful extending action of the ECRL on the first and second metacarpal, mediated by the pulley-like arrangement on the groove, would push both metacarpals towards the ground and lever the bat's body into the air. A similar launching action is used by *Desmodus rotundus *which flexes its elongated thumbs to push off from the ground [37,39]. Notably, in *M. tuberculata *the proximal third of the second metacarpal is covered by padded toughened skin [also noted by [17]]. This type of skin also occurs on the dorsal carpal area – the surface on which *Mystacina *walks – suggesting that the broad, flattened proximal second metacarpal is also frequently in contact with the substrate. However, because the metacarpals are normally carried flexed and off the ground when *Mystacina *walks [1], it suggests an alternative locomotor role for metacarpal II, probably in launching.

### Humeral morphology in an early Miocene Australian mystacinid

Australian mystacinids are known from craniodental fossils from deposits ranging in age from 26 to 12 Ma (late Oligocene to middle Miocene) in South Australia, Queensland and the Northern Territory [2,6]. Two partial fossil humeri (Figure 1A–E) from early Miocene (c.20 Ma) sediments in the Riversleigh WHA, northwestern Queensland (18° 15' 35" S, 138° 06' 41" E) are the first postcranial remains referable to an Australian mystacinid. Collectively, the morphology, size, provenance and depositional association of the fossil humeri indicate that they are specifically referable to the early Miocene mystacinid *Icarops aenae*, known otherwise from dentaries and upper and lower teeth from the Riversleigh WHA (see Methods).

The fossil humeri have been identified as mystacinid because they exhibit the following suite of derived features shared only with *M. tuberculata* and the recently extinct NZ *M. robusta* (Figure 1F–L): distal articular surface more or less aligned with shaft of humerus and inclined laterally with respect to the long axis of shaft; non-spherical central capitulum; long distal spinous process well separated from the trochlea; broad separation between central and lateral capitulum; prominent (tall) trochlea; deep radial fossa; and deep, wide groove, and well-developed tubercle, proximal to the lateral epicondyle. They differ from *Mystacina* species in the following less-derived features: distal articular surface slightly less inclined laterally with respect to the long axis of the shaft resulting from the greater distal extent of the lateral capitulum and epicondyle; and supraepicondylar groove and associated tubercle proximal to the lateral epicondyle slightly less developed. They differ additionally from *M. robusta* in being approximately 20% smaller. The tip of the spinous process is broken off in both Australian fossil humeri; this tip appears to be more curved towards the trochlea and the medial profile more convex in *M. robusta* than in *M. tuberculata*.

The presence of a lateral supraepicondylar groove much like that in *M. tuberculata *suggests that the ECRL was similarly arranged in *I. aenae*. It can therefore be expected that *I. aenae *had similar capacities of powerful metacarpal extension. If this arrangement is related to levering the animal off the ground, as we suggest here, it would be a strong indication of terrestriality in *I. aenae*. Furthermore, although the tip of the spinous process is missing in both *Icarops *humeri, the position of its base indicates a medial position of carpal flexor muscles and *pronator teres *that corresponds to that seen in *M. tuberculata*. Thus, carpal flexion patterns in *I. aenae *were similar to those of *M. tuberculata*. Lastly, the lateral inclination of the humeroradial articulation, although less pronounced, is similar to that of *M. robusta *so that adaptation for a scuttling walk seems probable. In summary, the humeral morphology of *I. aenae *is strongly suggestive of at least facultatively efficient terrestrial locomotion.

## Discussion

### Origin of mystacinids and their dispersal to New Zealand

**Figure 4:**
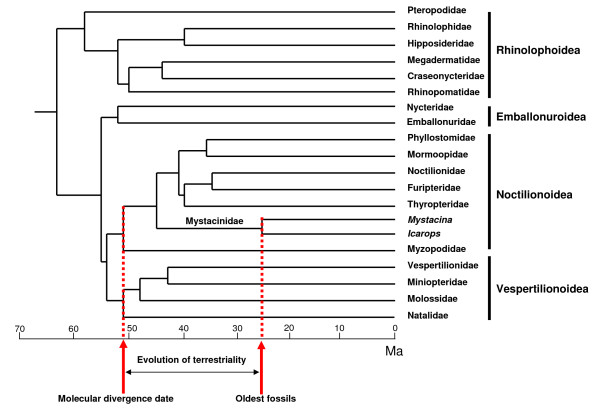
**Phylogenetic relationships and divergence times for extant bat families**. Phylogenetic relationships and estimates of divergence times for extant bat families after Miller-Butterworth *et al*. [40]. Terrestrial locomotion in the family Mystacinidae evolved sometime between 51 and 26 Ma.

Mystacinidae is a member of the superfamily Noctilionoidea, the only one of the four currently recognised extant bat superfamilies that appears to have a Gondwanan origin [[7,40]; however, see [41]]. Molecular divergence dates suggest that mystacinids diverged from other noctilionoids sometime between 41 and 51 Ma [e.g. [7,40,42]; [Figure 4]. A gap in the Australian land mammal record from 55 to 26 Ma [43] means that the early history of mystacinids in Australia is unknown, but the fossil record documents their presence as part of the indigenous fauna for at least 14 million years from 26 to 12 Ma. The oldest *Icarops *fossils are currently from the 26 Ma magnetostratigraphically-dated Ditjimanka Local Fauna (LF) of Lake Palankarinna, South Australia [28°46'30"S, 138° 24'E; ][6,44]. Although they are generally more plesiomorphic than Quaternary mystacinids, *Icarops *taxa also exhibit dental apomorphies of their own, suggesting that the Australian *Icarops *and NZ *Mystacina *lineages diverged at least 26 Ma [6]. Mystacinids are also known from the early Miocene (19–16 Ma) St Bathans Fauna of Central Otago, South Island, NZ (44°52'S, 169°49'E)[4,45] but as yet cannot be referred to either lineage.

If the molecular phylogenies and divergence dates for mystacinids are correct, and the highly derived humeral morphology shared by *Icarops *and *Mystacina *spp does reflect specialized terrestrial locomotory ability, this represents strong evidence that habitual terrestriality evolved in mystacinids sometime between 51 and 26 Ma (Figure 4).

The fossil record sheds little light on whether or not NZ might have been colonized before Australia by noctilionoids, but Australia's Oligo-Miocene mystacinids are closely related to those of NZ and are less derived, which may be an indication that Australia was the source of NZ's mystacinids [6]. NZ began rifting from Gondwana c.82 Ma [46] but separation from eastern Australia of the (now mostly submerged) Zealandia continental fragment was not complete until formation of the Cato Trough near present New Caledonia c.52 Ma [47,48], with a distance of c.1600 km now separating the NZ part of Zealandia from the Australian mainland. Isolated, wind-assisted dispersals by bats from Australia to NZ have been recorded during historic times [9], and the ancestor of NZ's only other surviving endemic mammal, the vespertilionid bat *Chalinolobus tuberculatus*, probably made this crossing less than 2 Ma [49]. There are no records of bat dispersals in the opposite direction, against the westerly winds that have prevailed since establishment of the Antarctic Circumpolar Current c.35 Ma [50]. For NZ birds, a similar pattern of colonisation from Australia and rarity of "reverse traffic" is well-documented [e.g. [51]].

### Palaeoecology and extinction of Australian mystacinids

Our data suggest that the terrestrial habits of mystacinids, at least as expressed in specializations of the humerus, were established before isolation of the lineage in NZ – in Australia and in a time and place well-populated by terrestrial nocturnal predators. The latter included rat- to cat-sized marsupial carnivores such as dasyures (dasyurids), cat- to leopard-sized marsupial lions (thylacoleonids), cat- to dog-sized thylacinids, dog-sized carnivorous/omnivorous kangaroos (species of *Ekaltadeta*), mouse- to cat-sized bandicoots (yaralids) and large predatory bats such as megadermatids, as well as hawks, terrestrial crocodiles, lizards and snakes [[43]; see Methods below].

In NZ, too, at least one group of small terrestrial mammals was present until at least the early Miocene, overlapping temporally and geographically with mystacinids [as recorded in the 19–16 Ma St Bathans Fauna; [5]], and so provides further evidence that the evolution of terrestriality in mystacinids did not arise in the absence of non-volant mammals in NZ.

Although it is likely that terrestrial foraging by extant *M. tuberculata *makes it vulnerable to introduced mammalian predators [e.g. feral cats and stoats; [11,52,53]], there is as yet limited available data on the actual risk of terrestriality. Indeed, Lloyd [8] has argued that although some individuals may be caught while on the ground, mystacinids would not be easy prey – they are cryptic, fast moving, with acute hearing and sense of smell, and can quickly take flight [8]. Based on the evidence presented here, it seems reasonable to assume that *Icarops aenae *was similar to *M. tuberculata *in these respects. The slightly less-developed morphological humeral specializations in at least *I. aenae*, one of four known Australian mystacinid species, suggest that the terrestrial habits of mystacinids may have further developed in NZ in the absence of terrestrial mammals but in the presence of falcons, moreporks and laughing owls which were significant predators [54].

In NZ, *Mystacina*'s terrestrial foraging behaviour has been correlated with its exceptionally broad omnivorous diet [9,11,14,15] that is broader than that of any bat recorded and includes nectar, flowers and fruit as well as flying and terrestrial invertebrates including spiders, centipedes and weta orthopterans [9,52,55,56]. An omnivorous diet in Australian Miocene mystacinids, and in particular *I. aenae*, has been been deduced on the basis of craniodental features [6]. For example, the dilambdodont molars of *Mystacina *species and *I. aenae *are typical of insect-eating bats, while their anterior teeth indicate adaptations for both frugivory and nectarivory [57,58]. Like other chiropteran frugivores, *Mystacina tuberculata *and *I. aenae *have a greater allocation of tooth area at the anterior end of the tooth row (individually large teeth accounting for half the upper tooth row length) than in more insectivorous and carnivorous species. They also share a reduced number of lower incisors, a fused mandibular symphysis and large canines, features that act together to support a quickly-moving, extensible tongue in nectar-feeding bats [58].

*Mystacina tuberculata *roosts singly or communally in tree hollows, but also uses its teeth to burrow into fallen trees to excavate complex roosts [9,15]. It is also known to have inhabited caves along with *M. robusta *at times during the last 15 000 years [59,60]. Large populations occur only in extensive (>1000 ha) areas of old-growth indigenous forests dominated by species of *Podocarpus*, *Dacrydium*, *Agathis *and *Nothofagus *and containing many large trees suitable for such roosts (>1 m girth and >25 m high), numerous epiphytes and deep leaf-litter [8]. The palaeohabitat of *Icarops aenae *appears to have been similar: 20 Ma the Riversleigh forests in which it foraged were c.15 degrees further south than the present fossil sites. The Australian climate was cooler and wetter with extensive cover of Gondwanan forests dominated by species of *Nothofagus *(*Brassopora *type), podocarps, araucarians, myrtaceans and casuarinaceans [61,62].

What caused the extinction of mystacinids in Australia is not clear but available evidence suggests that it was probably loss of suitable forest habitat resulting from climate change. Australian Tertiary mystacinids range in age from c.26 to 12 Ma [2,6,63], but they are absent from the diverse bat faunas of Riversleigh's early late Miocene Encore LF (c.10 Ma; 12 spp) and early Pliocene Rackham's Roost LF (c.4.5 Ma; 10 spp) [43]. By the late Miocene, mystacinids seem to have disappeared from Australia [2,63], perhaps as a result of cooling temperatures and reduced rainfall which began in the mid to late Miocene. This climatic change resulted in gradual replacement of wet Gondwanan-type forests by relatively drier forests, woodlands and, by mid Pliocene time, grasslands over much of the continent. Closed forests retreated to the coastal margins of Australia and it is possible that mystacinids survived there during the late Miocene but they are not known from Plio-Pleistocene bat faunas in those areas including, for example, the early Pliocene Hamilton, Chinchilla and Bluff Downs LFs and the Pliocene Big Sink LFs, nor from the Mount Etna, Texas, Bucchan, Victoria Fossil and Mammoth Cave assemblages [64,65]. Increasingly rapid cycles of climate change resulted in pronounced post-Miocene rainforest contraction and expansion, resulting in further biodiversity loss: the Mystacinidae is one of 10 mammal families lost from Australian rainforests (representing a decrease of 37% in mammalian familial diversity) since the early to middle Miocene [66].

## Conclusion

Australian Oligo-Miocene fossils suggest that the specialized terrestrial locomotion of mystacinids did not develop in NZ in the absence of ground-dwelling mammalian predators and competitors, but that facultatively-terrestrial behaviour in Gondwanan mystacinids may represent an exaptation for exploiting a predator-"free" nocturnal terrestrial niche in NZ. Mystacinids appear to have had a long history as omnivores on Gondwanan forest floors where mammalian predators and potential prey were plentiful [43].

In NZ, mystacinids found refuge in cool wet rainforests, surviving there for at least 16 Ma (other colonizing bat lineages did not fare as well; Hand *et al*. in prep.). The slightly less-developed morphological humeral specializations in at least *I. aenae *suggest that the terrestrial behaviours of mystacinids may have further developed in NZ, though in the presence of avian predators and at least one lineage of now-extinct non-volant mammals.

Our data suggest that the evolution of terrestrial behaviour in mystacinids may have been driven by selective advantage or energetic benefit, as has been proposed for the vampire *Desmodus rotundus *[1]. There is little doubt that the exceptionally diverse diet of *M. tuberculata *has been facilitated by its extraordinary terrestrial foraging and burrowing behaviour [9,11,14,15]. Australia's *Icarops aenae *also appears to have been diversely omnivorous [6]. An opportunistic approach to feeding may have conferred a selective advantage for bats inhabiting the high latitude forests of early Cenozoic Gondwana as well as their survival during later glaciation events in NZ. On-going studies of the seasonal foraging ecology and energy budget of *M. tuberculata *[e.g. [67-70]] may provide further clues about the driving forces underlying terrestriality in mystacinids, as well as any likely trade-off between aerial and non-aerial locomotion in bats that walk.

In New Zealand, during the last 750 yrs since the arrival of people [71], forest cover has been reduced from 78 to 23%, and 31 alien mammal species have become established [72]. These major ecological changes have affected *Mystacina *species directly by increasing predation and competition and indirectly by transforming remnant forest ecosystems [73]. These pressures led to the extinction of *M. robusta *within the last 50 years and a precipitous decline in the population of *M. tuberculata *from an estimated 12.5 million (pre-human) to c.50 000 today [73]. In the case of *M. robusta*, mainland populations appear to have declined rapidly following introduction of Pacific rats (*Rattus exulans*) by Polynesians [60], although the last known populations on Big South Cape Island and adjoining Solomon and Pukaweka Islands were exterminated following the accidental introduction of ships rats (*Rattus rattus*) in 1964 [74].

For Australia, there is no direct evidence of any mammalian extinctions coinciding with its first colonization by murids c.5 Ma [75,76], although there is speculation that the demise of the extinct frugivorous-omnivorous marsupial ektopodontid and numbigilgid lineages may have resulted [77,78]. The available fossil record suggests, however, it is unlikely that mystacinids survived in Australia long enough to overlap with rodents.

In the Pacific and globally, bats have reached most oceanic islands, the insectivorous hoary bat *Lasiurus cinereus *of the Americas, for example, having colonised the Galápagos and Hawaiian Islands [79]. Unlike in birds, where flightlessness has evolved independently many times in island taxa, such as in Pacific rails [20], there remains no evidence of loss of flight in any extinct or extant bat.

## Methods

### Systematic palaeontology

#### *Icarops aenae *[2]

##### Holotype

QM F30567, edentulous mandible preserving fragment of left dentary with alveoli for i1, c1, p2,4, m1–3 and right dentary fragment with alveoli for i1, c1, p2,4

##### Type locality

Wayne's Wok Site, Riversleigh World Heritage Area (WHA), Lawn Hill National Park, northwestern Queensland [66].

##### Additional material

QM F30584, left dentary containing m2 and m3; QM F24509, right m1; QM F30575, left M1; all from type locality [6]. A left dentary containing m2 and m3, with alveoli for c1, p3, p4 and m1, and an isolated left M1, recovered from Outasite in the Riversleigh WHA, may also represent this species [*Icarops *sp. cf. *I. aenae*; ][6].

##### New material

QM F30573, fragment of distal end of left humerus (Wayne's Wok Site); QM F30574, fragment of distal end of right humerus (View Delightful Site, Riversleigh WHA); Figure 1A–F.

##### Diagnosis

Humerus similar in basic morphology to that of Quaternary mystacinids *Mystacina robusta *and *M*. *tuberculata *but differs in lateral extension (development of tubercle) of proximal rim of lateral epicondyle, and less inclined distal articular surface (with respect to humeral shaft).

The morphology and provenance of the humeral remains indicate that they are referable to the Australian Miocene mystacinid genus *Icarops *(see Comparisons below). At least two *Icarops *taxa are represented in Riversleigh sediments (*Icarops aenae *and *I*. *paradox*) and another from Bullock Creek in the Northern Territory (*I*. *breviceps*) [2,6,63]. An isolated upper molar from Lake Palankarinna, northeastern South Australia [mystacinid indet.; [6]] also appears to be referable to *Icarops*. Other bats represented in Riversleigh's Oligo-Miocene deposits are: diverse and abundant hipposiderids (>22 spp), megadermatids (>5 spp), and much rarer remains of molossids (2 spp) and vespertilionids (1 sp.) [43]. The morphology of the distal humeri described here differs distinctly from that found in all other bat families [e.g. [28] Figure 2] except Mystacinidae (see Description and Comparisons below), and the specimens are most parsimoniously referred to Riversleigh's only known mystacinid genus, *Icarops*. Comparison with skulls and skeletons of Recent *Mystacina *spp indicate that the humerus is also an appropriate size to be attributed to a species of *Icarops*.

The fossil humeral remains are specifically attributed to *Icaops aenae *because of the depositional association of the humeral fragment QM F30573 with *I. aenae *craniodental remains (QM F30584, a left dentary containing m2 and m3, and QM F30575, an M1) preserved less than 15 cm away in a hand-sample of limestone collected from Wayne's Wok Site in which the dentary was partially exposed. This association is particularly noteworthy in a fossil deposit in which bat remains are relatively common (>1000 specimens) but non-hipposiderids rare (less than 0.5%). QM F30574, a left humeral fragment from View Delightful Site, is of the same morphotype and size as QM F30573 and is therefore also referred to *Icarops aenae*.

##### Locality and age

Wayne's Wok Site occurs on the western edge of freshwater limestones comprising Hal's Hill, and View Delightful Site on the southern edge of Godthelp Hill, both part of the D Site Plateau, Riversleigh WHA, Lawn Hill National Park, Queensland, Australia [64,80]. On the basis of Riversleigh stratigraphy and faunal assemblages, the Wayne's Wok and View Delightful deposits have been interpreted to be early Miocene in age [66,80-83].

##### Associated fauna and palaeoenvironment

The associated fauna from Wayne's Wok Site includes lungfish, teleost fish, frogs, chelids, scincids, agamids, pythonids, typhlopids, crocodylids, birds, pilkipildrids, acrobatids, petaurids, pseudocheirids, burramyids, ektopodontids, phalangerids, macropodids, potoroids, wynyardiids, diprotodontids, palorchestids, yalkaparidontids, dasyurids, thylacinids, perameloids, notoryctids, megadermatids and hipposiderids. The high diversity of vertebrates represented in this deposit, together with the fact that it contains complete skulls of marsupials but only fragmentary bat material, suggests that these fossils probably accumulated in a pool or lake rather than a cave. The fauna of the VD deposit is as yet poorly-sampled but includes a burramyid, koala and palorchestid [43] as well as hipposiderid bats. Archer *et al*. [84] give reasons for regarding early Miocene assemblages at Riversleigh to represent closed forest communities.

### Description

The following description of the humerus is based on both specimens (QM F30573 and QM F30574) except where indicated. Terminology for humeral morphology and orientation is given in Figure 1M–O and follows previous workers [28,32,38]. The shaft is only just wider than deep (anteroposteriorly), and the distal part of shaft is only slightly flattened anteroposteriorly and curved in a cranial direction. In QM F30573 the maximum width of the articular surface is 3.2 mm and that of the shaft 2.0 mm; in QM F30574 it is 3.1 mm and 2.0 mm respectively. The articular surface is slightly offset with respect to the shaft of the humerus, so that in anterior view (Figure 1A, D) both the trochlea and the lateral epicondyle rim are lateral to the edges of the shaft. The articular surface is inclined with respect to the long axis of the shaft (i.e. it is not perpendicular to the shaft) so that the trochlea is taller than the lateral epicondyle. The medial process (epitrochlea) is relatively narrow, in width approximately one-quarter that of the articular facets. The distal spinous process is well-separated from the trochlea. Its tip is missing in both specimens but clearly would have extended distally beyond the trochlea. The trochlea, central capitulum and lateral epicondyle are all prominent, the trochlea most prominent. The central capitulum is non-spherical and occupies less than one-third the width of the articular surface. The central and lateral surfaces of the capitulum are separated by the capitular groove, and the capitulum and trochlea separated by the trochlear groove. The trochlear and capitular grooves are equally deep and slightly inclined in a lateral direction. The trochlea's medial margin is concave, a ridge extends proximally along the shaft to enclose medially the deep and broad radial fossa. Posteriorly, the trochlear ridge extends proximally onto the shaft for a short distance (Figure 1C, F). A broad, shallow longitudinal depression is bounded laterally by a ridge that extends 2 mm proximally along the shaft. There is no depression for articulation with the ulnar olecranon process. At each end of the articular surface is a deep fossa. The medial fossa is bordered by the trochlear rim anteriorly and posteriorly by the lateral rim of the medial process (epitrochlea). The lateral fossa is surrounded by the rim of the lateral epicondyle. Immediately proximal to the rim is a broad and deep groove that extends across the lateral shaft and onto the posterior face of the shaft. This lateral supraepicondylar groove is bounded by a raised rim (almost as distinctive as the lateral epicondyle rim) that is marked proximally by a large tubercle (Figure 1B, E).

### Comparisons

The Riversleigh fossil humeral fragments share morphological features with many bat families. A non-spherical central capitulum and articular surface more or less aligned with the shaft are characteristics of molossids, vespertilionids, miniopterids, mystacinids, mormoopids and rhinolophids. In archaeonycteridids, most emballonuroids, most other noctilionoids, hipposiderids and pteropodids, the capitulum is spherical and articulation conspicuously offset laterally with respect to the shaft. In myzopodids the articulation is aligned but the capitulum is spherical.

In the Riversleigh fossil specimens there is a broad separation between the central and lateral capitulum, as in molossids, mystacinids and miniopterids, and unlike most other bat groups [e.g. see [28] Figures 3 to 5, and [85] Figures 7 to 12]. The fossils differ from miniopterids in lacking a deep groove distally between the central and lateral capitulum. The Riversleigh specimens also lack the conspicuous depression for articulation with the ulna on the posterior surface that is exhibited by most vespertilionids and palaeochiropterygids.

The medial process (epitrochlea) is wider than in most vespertilionids and molossids, but not as wide as in palaeochiropterygids, archaeonycteridids, rhinolophoids, emballonuroids, natalids, most noctilionoids and pteropodids; it is similar in relative width to that in mystacinids, myzopodids and furipterids. The distal spinous process is well separated from the trochlea, as in most bat families, but not molossids and vespertilionids. It is also relatively long (extending distally beyond the trochlea) as in many bat groups.

The radial fossa is deep and the distal part of the shaft is slightly flattened as in mystacinids, molossids and vespertilionids but not most other bats. Lateral and medial fossae are deep, and the trochlea prominent, as they are in molossids and mystacinids but not most other bats.

The lateral supraepicondylar groove or depression developed proximally to the tubercle of the lateral epicondyle is broader and deeper with a more conspicuous rim than in other bats, with the exception of mystacinids in which, as in the Riversleigh specimens, it occupies an area equal to that of the lateral (epicondylar) fossa. In other bat families in which the groove may occur, such as molossids and vespertilionids, it is conspicuously shallower and smaller in area [e.g. [32], Figure 6, [85], Figures 7 to 12].

As noted above in **Results**, the humeral fossils exhibit several derived features shared only with *M. tuberculata *and *M. robusta *(Figure 1G–L).

## Authors' contributions

SJH carried out the morphological comparisons, analysed and interpreted the data and drafted the manuscript. VW carried out the dissection, interpreted the anatomical data and helped draft the manuscript. RMDB interpreted the phylogenetic and biogeographic implications of the data and helped draft the manuscript. MA interpreted the palaeontological, geological and palaeoecological data and helped draft the manuscript. HG collected and identified the fossil specimens, provided geological data and helped draft the manuscript. AJDT and THW interpreted the palaeoecological and biogeographic data and helped draft the manuscript. All authors read and approved the final manuscript.
